# Foot-and-mouth disease virus-derived dendrimer peptides induce germinal center-dependent antibody responses and IgG1 plasma cell differentiation in mice

**DOI:** 10.1038/s41598-026-42982-2

**Published:** 2026-03-12

**Authors:** Marta Iborra-Pernichi, Patricia de León, Elisa Torres, Sira Defaus, Esther Blanco, David Andreu, Nuria Martínez-Martín, Francisco Sobrino

**Affiliations:** 1 Centro de Biología Molecular “Severo Ochoa” (CSIC-UAM), 28049 Madrid, Spain; 2 Centro de Investigación en Sanidad Animal (CISA; INIA-CSIC), 28130 Valdeolmos, Madrid, Spain; 3 Department of Medicine and Life Sciences, Universitat Pompeu Fabra, 08003 Barcelona, Spain; 4 Intestinal Morphogenesis and Homeostasis Group, Area 3-Cancer, Instituto Ramón y Cajal de Investigación Sanitaria (IRYCIS), Madrid, Spain

**Keywords:** FMD, FMDV, B cell response, T dependent, Germinal center, Peptide-vaccines, Biotechnology, Immunology

## Abstract

Our previous findings demonstrated that the B_2_T dendrimer peptide, harboring B- and T-cell foot-and-mouth disease virus (FMDV) epitopes, confers protection in swine and elicits in mice significant levels of neutralizing antibodies, a key correlate of protection against FMDV. Here, we use a mouse model to study the germinal center (GC) B-cell response in B_2_T-immunized mice, as this peptide is aimed to enhance antigen-specific immune responses, relying on T-cell help to potentiate B-cell activation and maturation. Flow cytometry revealed that immunization with B_2_T enhanced GC formation and increased the proportion of IgG1-positive GC B-cells in draining lymph nodes. Additionally, elevated levels of IgG1-secreting B-cells in bone marrow are consistent with the initiation of GC–derived plasma cell differentiation, highlighting B_2_T’s ability to potentiate antigen-specific immunity through coordinated B- and T-cell activation. The study demonstrated that only B_2_T, but not the version lacking T-cell epitope (B_2_), triggered neutralizing antibody production and a robust T-cell response in the spleen, underscoring the critical role of CD4^+^ T-cell help mediated by the T-cell epitope in eliciting protective immunity. Even though these studies need to be extended to natural hosts such as swine, our results highlight the role of the B_2_T dendrimer in inducing IgG1-secreting long-lived plasma cells in the bone marrow, providing insights into the mechanisms underlying the durable immunity elicited by epitope-linked dendrimer vaccines.

## Introduction

Foot-and-mouth disease (FMD) is a highly contagious viral infection caused by foot-and-mouth disease virus (FMDV), which primarily affects cloven-hoofed animals such as cattle, pigs and sheep. It represents a major threat to the livestock industry and is recognized as a notifiable disease by the World Organization for Animal Health (WOAH)^1^. FMD is endemic in various regions, particularly in parts of Asia and Africa, and is currently managed through the use of inactivated virus vaccines. However, these vaccines present notable limitations, including the risk of viral release during production and the inability to distinguish between infected and vaccinated animals through serological tests. Furthermore, controlling FMD in endemic areas remains a challenge, often resulting in substantial economic losses due to trade restrictions^2^. FMD can also suddenly re-emerge in disease-free territories; the recent outbreaks (January–April 2025) of the disease in Germany^3^, Hungary and Slovakia^4^ illustrate the susceptibility to this disease in even highly-monitored areas of the world.

To address these challenges, there is a pressing need for novel and more effective vaccine strategies. An essential aspect of protective immunity against FMDV is the generation of neutralizing antibodies (nAbs), which prevent the virus from attaching to integrin receptors on host cells and facilitate viral clearance through mechanisms such as virion disassembly^5^.

B cells, as central components of the adaptive immune system, are responsible for antibody production and the generation of a memory compartment. Upon encountering antigen, B cells can be activated through T-dependent or T-independent pathways. In viral infections like FMD, T-dependent B cell responses are predominant, resulting in the production of high-affinity neutralizing antibodies^6^. This occurs via the formation of transient structures called germinal centers (GCs) within secondary lymphoid organs. Following antigen encounter and interaction with CD4^+^ T-helper (Th) cells, B cells differentiate into short-lived plasma cells (plasmablasts) and memory B cells during the extrafollicular response. Additionally, a subset of B cells migrates to the follicular center to establish GCs, where they undergo somatic hypermutation (SHM) and affinity maturation, ultimately giving rise to long-lived plasma cells (LLPCs) that home to the bone marrow (BM), and memory B cells (MBCs) that provide long-term immunity^7^. Given the critical role of GCs in generating long-lasting, high-affinity antibody responses, vaccine strategies that actively promote GC formation are highly desirable to ensure robust and durable immunity.

Peptide-based subunit vaccines, particularly those based on multiple antigen-presenting systems (MAPs)^8^, have emerged as advantageous alternatives to conventional formulations. Our recent efforts have showcased peptide-based platforms that elicit robust B- and T-cell responses, leading to the induction of neutralizing antibodies and broader protection against diverse FMDV strains^9–13^. In this context, our B_2_T prototype—encasing two copies of the B-cell epitope from VP1 (140–158) and one T-cell epitope from protein 3A (21–35)—has emerged as a safe, cost-effective candidate, conferring protection in swine, a natural FMDV host, with a single-dose immunization and eliciting consistent and long lasting humoral and cellular responses^9,10,13,14^. Moreover, studies on swine immunized with B_2_T have revealed a significant correlation between specific SLA-II haplotypes and T-cell responses, as well as a weaker correlation with antibody responses^15^. Dendrimer-specific recall responses included IFN-γ-producing CD4^+^ memory T cells and cytotoxic CD8β^+^ T cells^16^.

Despite these promising findings, the extent to which B_2_T induces GC responses and promotes the generation of neutralizing antibodies through long-lived memory remains unclear. This study aims to characterize the immune response elicited by B_2_T, focusing on GC formation and the development of durable humoral memory.

To this end, we employed outbred Swiss ICR1-CD1® mice as a cost-effective and genetically diverse surrogate model that allows the evaluation of immune responses in a genetically heterogeneous population that better mimics the diversity of natural FMDV hosts^17^. Our previous work demonstrated that B_2_T induces robust neutralizing antibody responses in CD1 mice^18^ and that co-delivery of B- and T-cell epitopes on a single molecular scaffold is essential for effective antibody induction^19^. In the present study, we evaluated B cell responses in the draining lymph nodes, spleen, blood, and BM following B_2_T immunization.

Flow cytometry analysis revealed enhanced GC formation and an increased frequency of IgG1^+^ GC B cells, particularly in draining lymph nodes. Furthermore, we observed a notable rise in IgG1-secreting B cells in the BM, consistent with the differentiation of class-switched, GC–derived plasma cells in the bone marrow. These findings underscore the potential of the B_2_T dendrimer vaccine to generate GC-dependent, IgG1-producing LLPCs, offering key insights into the mechanisms behind durable immunity induced by epitope-linked dendrimer vaccines.

## Results

### B_2_T immunization of CD1 mice induces specific humoral and cellular adaptive immune responses in draining lymph nodes

The adaptive immune response against FMDV involves humoral and cellular components, both essential for the protection against the disease. We first confirmed, as previously demonstrated^18,19^, that B_2_T (Table 1) immunization induced FMDV-specific humoral response.

**Table 1:**
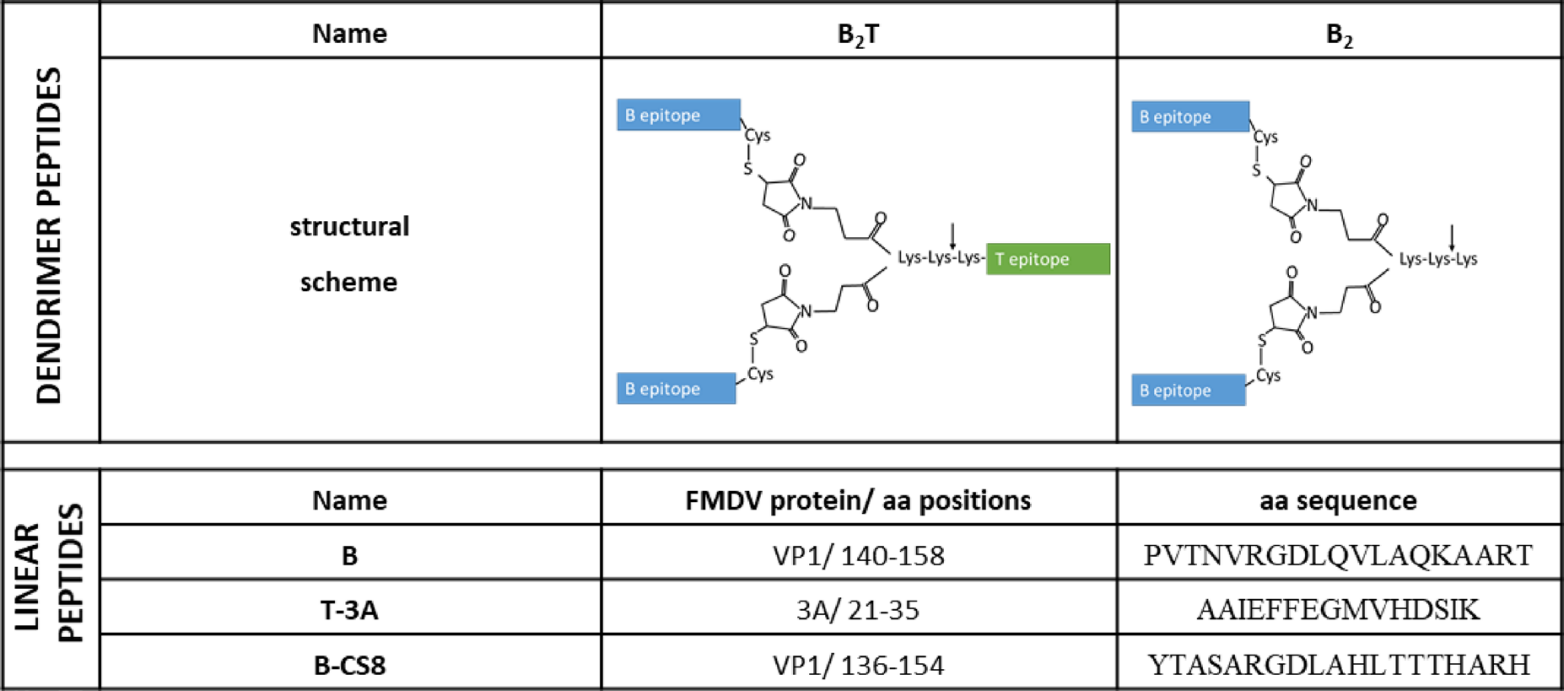
Dendrimer peptides used as immunogens. Vertical arrow denotes a putative cleavage site for cathepsin D, a protease involved in in vivo processing for MHC class II^13^. Position and amino acid sequence of linear peptides are displayed.

**Fig. 1:**
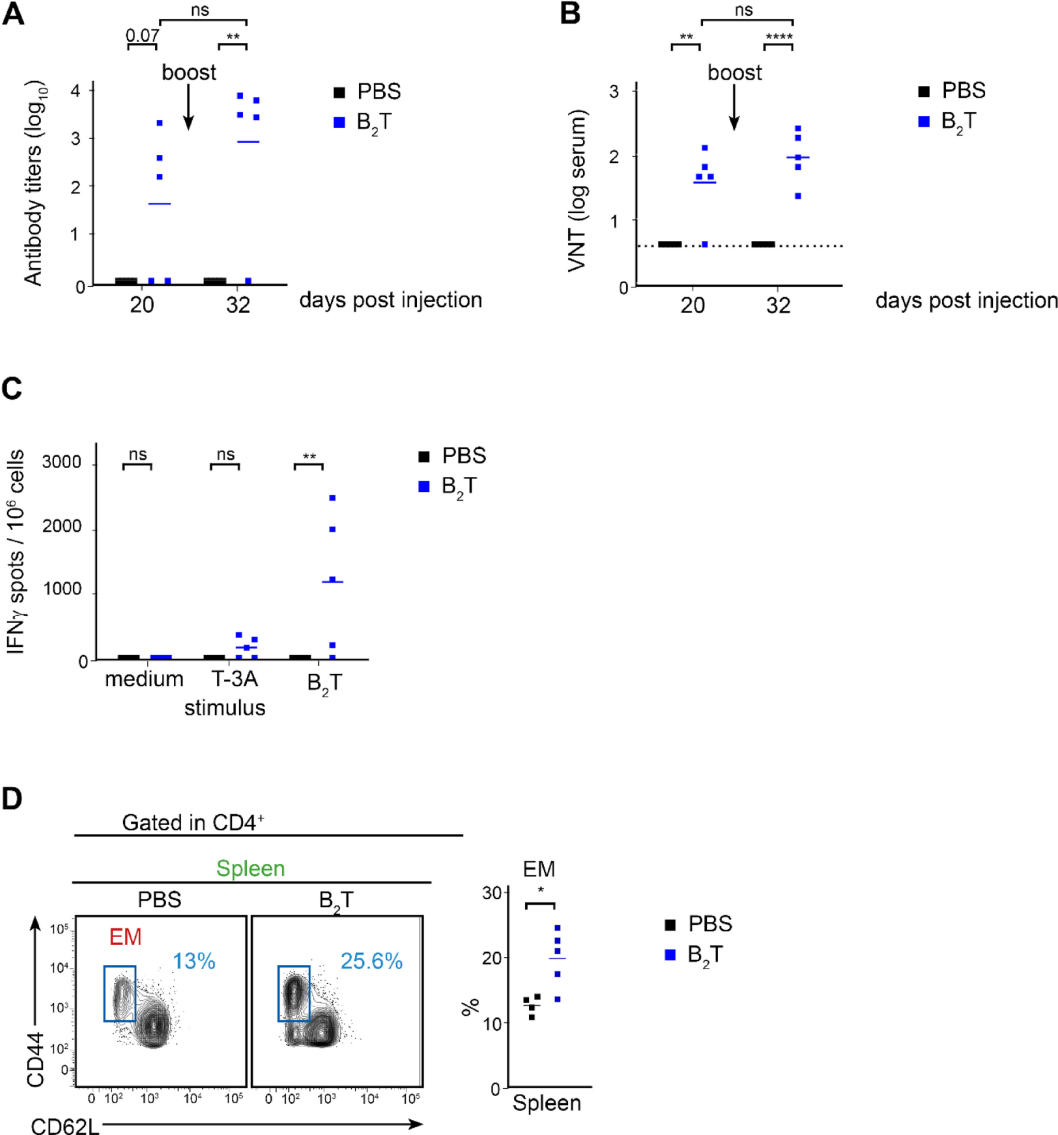
FMDV-specific humoral and cellular response induced in mice following dendrimer immunization (**A**) Total IgG antibodies against B peptide were detected by ELISA from sera of immunized mice at 20 dpi and 32 dpi. (**B**) Virus neutralization titers are expressed as the reciprocal log_10_ of the last serum dilution that neutralized 100 TCID_50_ of homologous FMDV. The dotted line indicates the detection limit. Each point represents the mean of triplicate values from an individual animal and horizontal lines indicate the mean of each group. Arrows in (**A**) and (**B**) indicate the booster immunization at 20 dpi. (**C**) IFN-γ ELISPOT at 32 dpi. Mice splenocytes were stimulated in vitro with B_2_T or with T-3A. The frequency of FMDV-specific IFN-γ secreting cells was determined as detailed in experimental section. Each point represents the mean of triplicate values from an individual animal. Horizontal lines indicate the mean of each group. (**D**) Left, representative flow cytometry plot showing splenic effector memory CD4^+^ T cells (gated as CD44^+^, CD62L^-^) within CD4^+^ population (32 dpi). Right, corresponding bar chart indicating the proportion of effector T cells. Each dot represents one mouse. For all panels unpaired two-tailed t-test was conducted. *P* values: *p < 0.05, **p < 0.01, ****p < 0.0001.

To do so, groups of five mice were immunized and boosted at day 20 post-immunization (20 dpi) with B_2_T or with PBS as control (in both cases with adjuvant). Sera were collected and total IgG antibodies targeting the B peptide were assessed by ELISA at 20 dpi and at 32 dpi (12 days post-boost) (Fig. 1A). The primary immune response (20 dpi) already showed a significant production of IgG antibodies compared with PBS. More importantly, mice response after the boost (32 dpi) was stronger and more robust than at 20 dpi, with a pronounced increase in IgG titers reflecting a successful recall.

Neutralizing antibodies are one of the more effective mechanisms of controlling FMDV spread^20^. Therefore, sera from immunized mice were tested for neutralizing activity against the homologous virus FMDV O/UK/11/2001. At 20 dpi, nAb titers of B_2_T-immunized mice increased in the sera, significantly vs. the control group, similar to what was observed for IgG levels (Fig. 1B). Notably, at both 20 and 32 dpi, control animals showed neither IgGs nor nAbs, indicating the specificity of the immunization in neutralizing FMDV (Fig. 1A and B).

The cellular branch of the adaptive immune response is effected by T cells. For antiviral immunity, the production of pro-inflammatory cytokines such as IFN-γ is especially important. Thus, to evaluate the impact of B_2_T immunization in the cellular branch of the response, we analysed by ELISPOT the production of IFN-γ in splenocytes harvested from mice and stimulated with antigen (B_2_T or T-3A) at 32 dpi. B_2_T immunization significantly enhanced the number of IFN-γ secreting spots, except for two mice that did not respond to the same extent. Interestingly, and supporting specific T-cell activation, ELISPOT revealed significant levels of IFN-γ secreting cells mainly found upon in vitro recall with B_2_T and, to a lesser extent, with T-3A (Fig. 1C). These results highlight the effectiveness of the peptide-based strategy in inducing antigen-specific humoral and cellular responses consistent with effective T-dependent recall capacity.

A major source of these cytokines is the CD4 effector memory (EM) T cells, characterized by their ability to rapidly produce cytokines upon antigen recall. When mice were immunized and boosted with B_2_T peptide, the amount of EM cells was significantly increased compared to control group, indicating a successful recall response, strictly dependent on the immunogen (Fig. 1D).

Overall, these data along with the antibody profile suggest that B_2_T induced a Th1 biased response, associated with protective immunity against FMD^5^.

### B_2_T immunization of CD1 mice induces specific GC response in draining lymph nodes

**Fig. 2:**
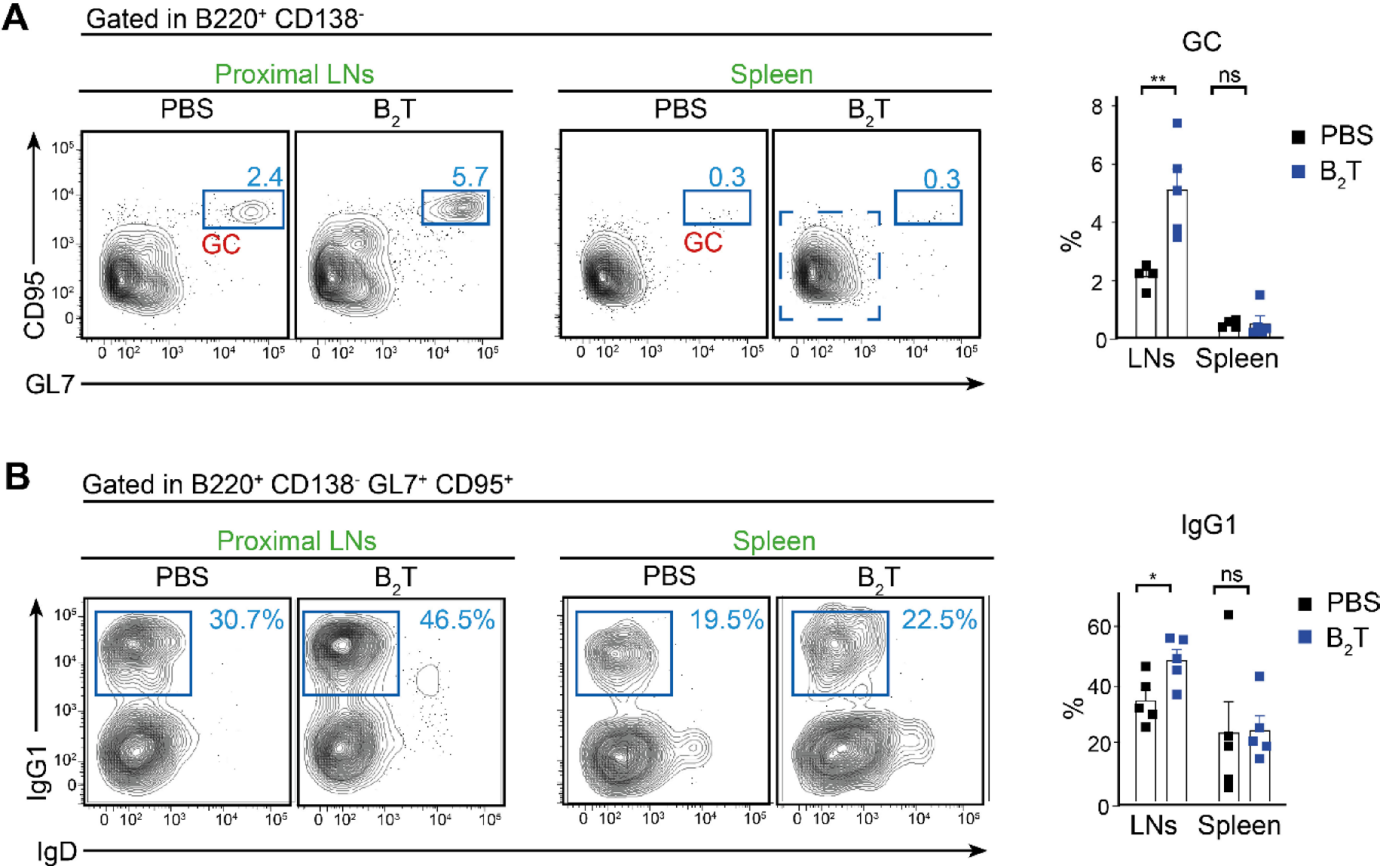
Germinal center analysis. (**A**) Left, representative FACS plot showing splenic and LNs total GC B cells (GC GL7^+^, CD95^+^) gated in B220^+^, CD138^-^ population (32 dpi). Right, bar chart indicating the proportion of GC B cells. (**B**) Left, representative FACS plot showing splenic and LNs specific IgG1^+^ cells within GC B cell population gated in (**A**) (32 dpi). Right, bar chart indicating the proportion of IgG1^+^-GC B cells. Each dot represents one mouse. For all panels unpaired two-tailed t-test was conducted. *P* values: *p < 0.05, **p < 0.01.

We aimed to investigate whether the strong and effective humoral immune response triggered by B_2_T correlates with the establishment of a GC reaction. FACS analysis performed in spleen and draining lymph nodes from mice boosted at 20 dpi with B_2_T dendrimer or with PBS revealed the formation of GCs detected as B220^+^, CD95^+^, GL7^+^ cells. At 32 dpi (12 days after the boost), the basal levels of GC cells were similar in spleen, while a statistically significant increase of GC B cells in draining LNs was observed upon B_2_T immunization (Fig. 2A). Moreover, the amount of IgG1^+^ GC B cells was significantly increased upon immunization in draining LNs, indicating the specificity of the immunization in promoting a robust humoral response elicited by B_2_T (Fig. 2B).

Overall, our data demonstrate the ability of B_2_T to induce a robust nAb production that correlates with the initiation of a GC response in draining lymph nodes, as well as an increase in class-switched IgG1-expressing B cells.

**Fig. 3:**
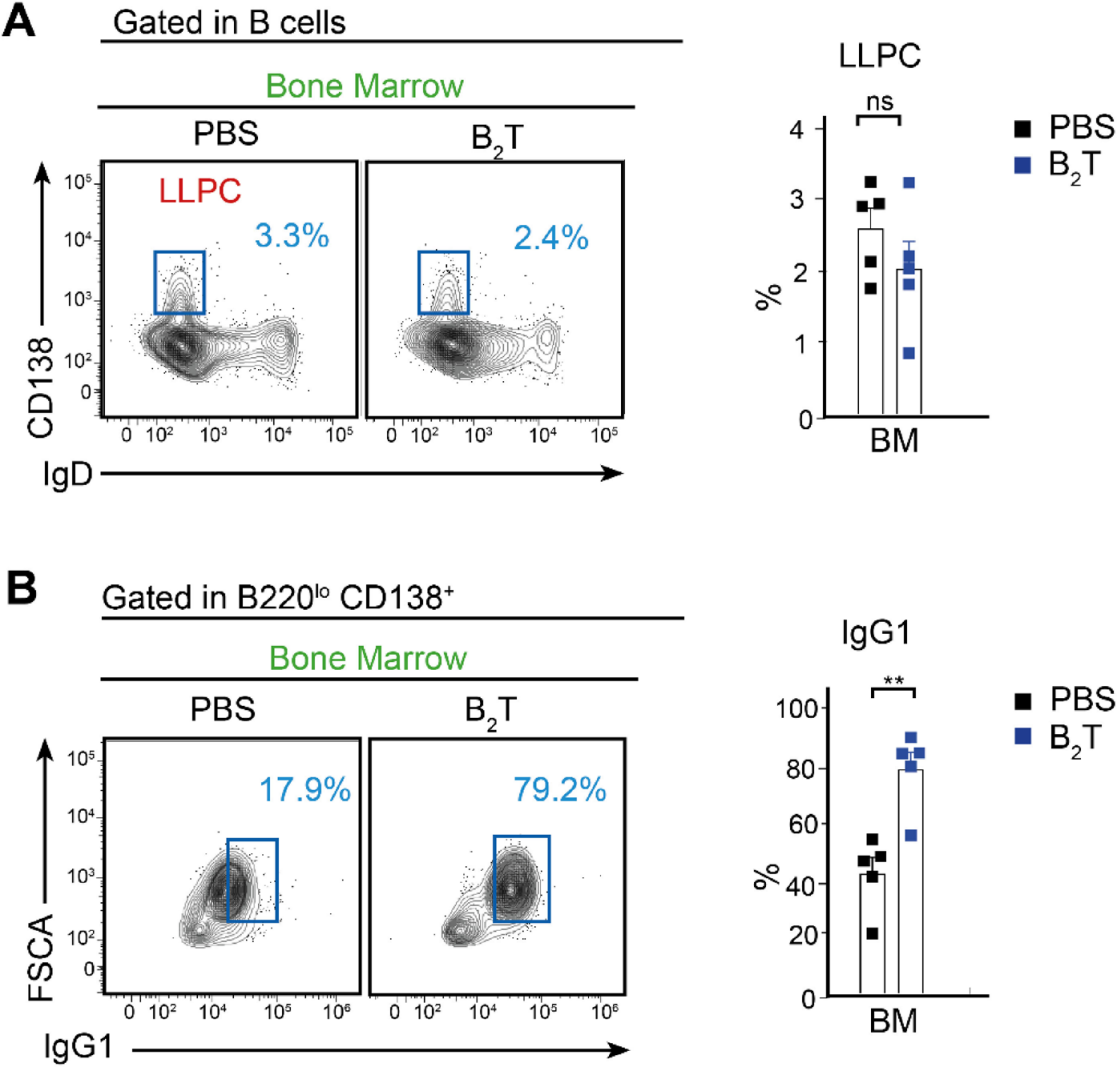
Bone marrow analysis. (**A**) Left, representative FACS plot showing BM LLPCs (B220^lo^, IgD^-^, CD138^+^) 32 dpi. Right, bar chart indicating the proportion of LLPCs. (**B**) Left, representative FACS plot showing specific IgG1^+^ LLPCs gated in (**A**) 32 dpi. Right, bar chart indicating the proportion of IgG1^+^ cells. Each dot represents one mouse. For all panels unpaired two-tailed t-test was conducted. *P* values: **p < 0.01.

The GC reaction leads to the generation of LLPCs, which migrate to the BM and provide a long-lasting source of neutralizing antibodies. We aimed to test the ability of B_2_T to trigger the differentiation of these effector cells. In the course of the humoral response, most of the plasma cells are short lived; however, a minor LLPC population can persist for longer periods of time^21^. In order to evaluate whether B_2_T immunization induced LLPC differentiation, we analyzed the BM compartment of immunized mice at 32 dpi, 12 days after the boost. Although the overall frequency of BM LLPCs (B220^lo^, IgD^-^, CD138^+^) did not differ significantly between groups, intracellular staining revealed a significant enrichment of IgG1^+^ LLPC in B_2_T-immunized mice, indicating a qualitative shift toward class-switched (GC-derived) plasma cells rather than a global expansion of the BM plasma cell compartment (Fig. 3), which correlated with elevated serum IgG titers (Fig. 1A).

Overall, our data supports the ability of B_2_T to prompt an effective T-dependent adaptive immune response, characterized by the triggering of GC reaction in draining lymph nodes.

### The uniqueness of B_2_T in triggering a T-dependent immune response underlies the effectiveness of the immunization

The above evidence suggests that B_2_T immunization triggers GC formation in draining lymph nodes, correlating with LLPC emergence and nAb production. To confirm whether this effect depends on a T-dependent mechanism, we compared the immunogenicity of B_2_T with that of B_2_, a branched construct preserving both B-cell but lacking the T-cell epitope (T-3A) (Table 1).

**Fig. 4:**
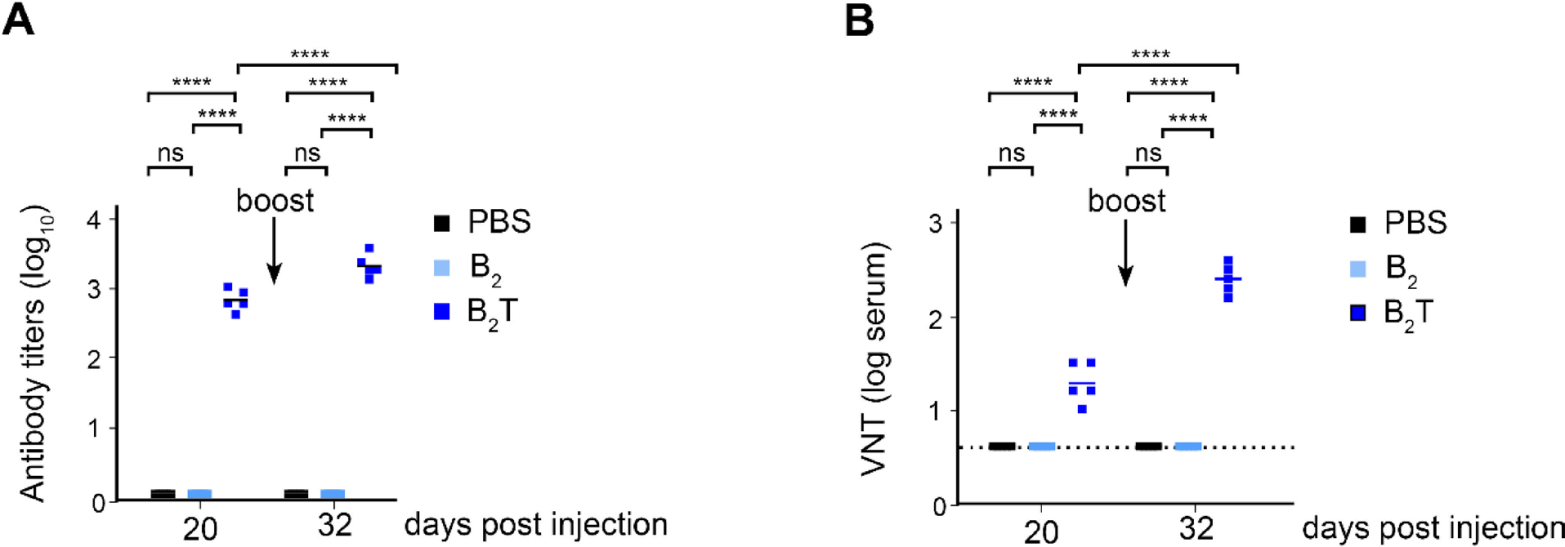
FMDV specific antibodies induced in mice following B_2_T and B_2_ immunization. (**A**) Total IgG antibodies against peptide B detected by ELISA from sera of immunized mice after the first (20 dpi) and the second peptide dose (32 dpi). (**B**) Virus neutralization titers are expressed as the reciprocal log_10_ of the last serum dilution that neutralized 100 TCID_50_ of homologous FMDV after the first and second dose. The dotted line indicates the detection limit. Each point represents the mean of triplicate values from an individual animal and horizontal lines indicate the mean of each group. The arrow within the figure indicates the booster immunization at 20 dpi. For all panels Two-way ANOVA was conducted with additional uncorrected Fisher’s LSD multiple comparison test was conducted. *P* values: ****p < 0.0001.

Groups of five mice were immunized with PBS, B_2_ or B_2_T (all with adjuvant), and blood samples were collected at 20 dpi (pre-boost) and 32 dpi (12 days post-boost). As previously observed, B_2_T elicited robust and specific anti-peptide IgG responses in all vaccinated animals, which were enhanced upon boosting. In contrast, B_2_-immunized mice failed to develop detectable antibody responses, same as for the PBS control group (Fig. 4A).

Virus neutralization assays confirmed that only B_2_T-immunized animals generated nAbs against FMDV O/UK/11/2001, with significantly increased titers following the booster dose (Fig. 4B). No neutralizing activity was detected in B_2_-inoculatedd mice, reinforcing the requirement for T-cell cooperation.

**Fig. 5:**
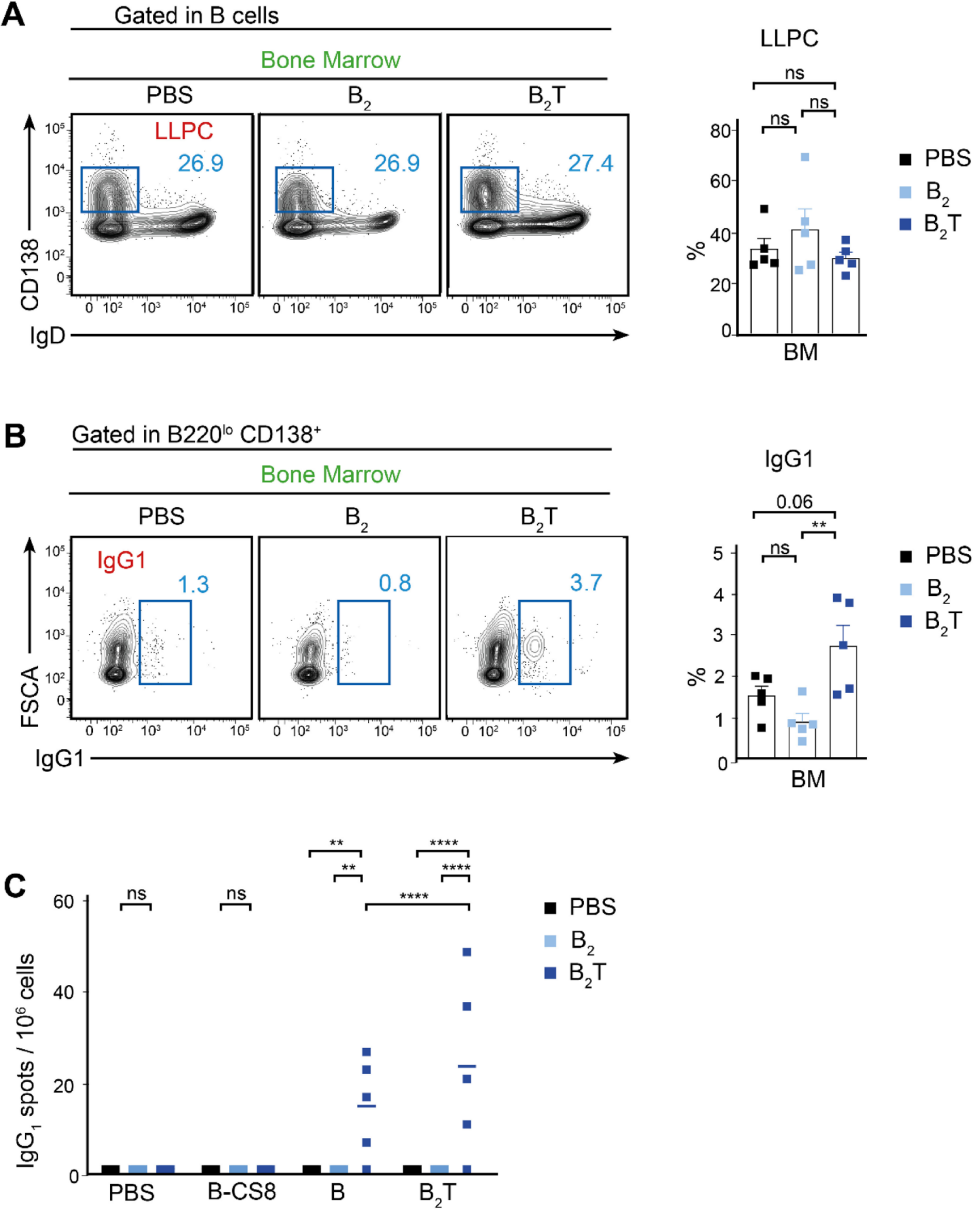
Evaluation of B cell response in the presence or absence of T epitope. (**A**) Left, representative FACS plot showing BM LLPCs (B220^lo^, IgD^-^, CD138^+^) 32 dpi. Right, bar chart indicating the proportion of LLPCs. (**B**) Left, representative FACS plot showing specific IgG1^+^ cells within LLPC B cell population gated in (**A**) 32 dpi. Right, bar chart indicating the proportion of IgG1^+^ cells. (**C**) B-cell ELISPOT. Different amounts of BM cells isolated at 32 dpi were shed in wells, previously coated with B_2_T, homologous B peptide and heterologous B-CS8 peptide (from FMDV C-S8c1 strain), and the number of cells expressing IgG1 was measured; each point represents the mean of triplicate values from an individual animal and horizontal lines indicate the mean of each group. For panels A and B One-way ANOVA, with additional Tukey’s multiple comparisons test, and for panel C Two-way ANOVA with uncorrected Fisher’s LSD multiple comparison test, were conducted. *P* values: **p < 0.01, **** *p* < 0.0001.

To further evaluate the role of T-cell help in the establishment of IgG1^+^ LLPCs we assessed IgG1-secreting LLPCs in the BM at 32 dpi. While both B_2_T and B_2_ led to the presence of LLPCs (Fig. 5A), only B_2_T induced a significant population of IgG1-secreting cells, indicating that T-cell help is critical for establishing a specific memory compartment of IgG1-secreting cells (Fig. 5B).

Additionally, B cell ELISPOT assays were performed to quantify antigen-specific antibody-secreting cells (ASCs) in the BM after in vitro recall with either B_2_T or homologous B peptide. A heterologous B peptide (B-CS8), derived from FMDV type C isolate C-S8c1, served as a negative control (Table 1). The highest frequencies of IgG1-expressing plasma cells were observed in B_2_T-immunized mice, correlating with IgG1^+^ B cell recall responses to B_2_T and homologous B, but not to heterologous peptide (Fig. 5C).

Taken together, these results underpin the T-dependent nature of B_2_T immunogenicity. The absence of both nAbs and IgG1^+^ memory plasma cells in B_2_-immunized mice confirms that effective humoral immunity requires T-cell cooperation.

## Discussion

The B_2_T peptide has been shown to confer FMDV-protective immunity in swine, with substantial evidence suggesting that such protection is primarily mediated through the efficient induction of nAbs^14^. The present study sheds further light on the mechanisms underlying protection, underscoring the unique capacity of B_2_T to trigger a highly specific and efficient immune response. Understanding the molecular and cellular mechanisms that enhance immune response is essential for the rational design of effective vaccines, particularly those based on subunit immunogens. A key criterion for vaccine success is the induction of long-lasting antibody responses, which depend on a strong and sustained GC reaction^22^. For instance, studies in individuals immunized with the mRNA vaccine BNT162b2, encoding the spike protein from SARS-CoV-2, by direct GC B cell sampling, revealed that GCs could persist for at least 6 months after vaccination, enabling durable humoral immunity^23^. Similarly, a recent study demonstrated that a persistent GC response, evoked by an influenza vaccine, resulted in a higher frequency of vaccine-specific BM LLPCs and that clones emerging from these persistent GCs exhibited enhanced affinity for vaccine antigens, as well as the ability to bind and neutralize a variety of influenza virus strains^24^.

It is well established that adult mice show variable susceptibility to FMDV infection depending on both host and viral strain, as well as on experimental parameters such as the dose and route of inoculation^25^. Unfortunately, Swiss ICR1-CD1® mice exhibit very low susceptibility to FMDV infection^26^, which precludes a reliable challenge model in this strain. Conversely, although C57BL/6 mice are among the most susceptible mouse strains to FMDV infection, we and others have observed that the B_2_T construct does not elicit detectable FMDV-specific nAbs in this genetic background. For these reasons, the present study focused on a detailed immunological analysis rather than on challenge experiments in mice. Likewise, the inclusion of a control group, i.e. immunized with an inactivated FMDV vaccine, in future studies, will allow assessment of whether the immune response elicited by B_2_T is superior, equivalent, or inferior to existing vaccine platforms in terms of GC formation.

Our study demonstrates that B_2_T immunization promotes robust localised GC formation and the subsequent development of LLPCs, essential for sustained humoral immunity. High antibody titers or robust GC responses do not necessarily correlate with protective immunity, and thus in vivo challenge studies are required to confirm the protection of B_2_T in the mouse model. In any case, and regarding their use as field vaccines, the capacity of B_2_T dendrimers to confer consistent and long-lasting protection in swine has been reported^9–15^.

The development of strong GC responses that afford long-lasting immunity can be influenced by antigen avidity. For instance, in HIV vaccination, it has been shown that antigen multimerization significantly enhances the recruitment of naïve B cells into GCs relative to the monomeric form of the same antigen^27^. Other similar studies on HIV consistently showed that multimerization activated a broader range of cognate B cells, including those with lower initial affinity. In contrast, monomeric antigens tended to induce weaker B cell responses and preferentially recruited only high affinity B cells, limiting the diversity and magnitude of the GC reaction^28,29^. Likewise, our B_2_T vaccine candidate, with its combination of different epitopes, could somehow mimic the high-density, repetitive epitope presentation of successful viral immunogens, thereby enhancing BCR cross-linking and activation of a broader pool of naïve B cells, and promoting stronger and more diverse GC responses.

The durable immune response evoked by B_2_T is crucially dependent on the particular architecture of the dendrimer, where two copies of the B cell epitope are tethered to a T-cell epitope that enables effective CD4⁺ help for B cell activation. Such a layout is relevant in that, while B cell epitopes induce antibody production, T cell help is key for generating high-affinity, long lasting antibody responses and immunological memory. This need for joint B/T epitope presentations has long been established in a number of cases. For instance, peptide vaccines based on B epitopes from the hepatitis B virus surface antigen failed to elicit strong antibody responses unless a T helper epitope was included^30^. Similarly, recent work on SARS-CoV-2 demonstrated that vaccines lacking T cell epitopes resulted in suboptimal nAb titers and poor memory responses^31^. In our present case, stimulation of B cells in the absence of T-cell engagement—as found for the B_2_ control immunogen lacking the T epitope—fails to induce a response. This result confirms that the T cell peptide from FMDV protein 3A (21–35) encased in B_2_T can provide cooperation to potentiate B cell activation and maturation. Indeed, a B_2_T prototype combining the T-3A (21–35) peptide with CSFV B-cell epitopes improved antibody response against CSFV and high levels of protection against virus challenge^32^. In conclusion, our results underscore the essential role of both dendrimer architecture and T-cell-dependent mechanisms in shaping the quality and durability of the antibody response.

Studies in pigs showed that vaccination with serotype O FMDV killed vaccines, adjuvanted with the potent immunopotentiator CVC1302, elicited a long-lasting humoral immune response^33^. Consistent with this, our results show that B_2_T immunization was associated with increased GC activity in draining lymph nodes, notably marked by a higher proportion of IgG1⁺ GC B cells. Additionally, we observed a significant increase in IgG1-secreting B cell populations in the BM, indicating the establishment of a persistent humoral response. Furthermore, only B_2_T-immunized mice developed detectable levels of neutralizing antibodies and exhibited enhanced T-cell responses in the spleen. Remarkably, these immunological features were absent in animals immunized with the B_2_ control peptide, further underscoring the importance of T-cell epitope inclusion. Indeed, we have recently shown that B_2_T-like dendrimeric constructs displaying combinations of two different FMDV T-cell epitopes can modulate the efficient protective responses evoked in swine^16,17^.

Due to the limited availability of reagents for GC detection in swine, Swiss ICR1-CD1® mice were selected to investigate the mechanisms underlying GC responses and memory formation, as an initial step towards understanding long-term immune memory in the target species. Given the known interspecies differences in immune system function, future experiments are required to determine whether the GC response and memory formation observed in mice are conserved in swine.

Taken together, these findings advance our understanding of the molecular and cellular requirements for effective B cell activation by epitope-linked dendrimer vaccines. Specifically, we underscore the role of B_2_T in driving the differentiation of IgG1-secreting LLPCs in the BM, offering new insights into the mechanisms by which dendrimer-based platforms can elicit robust and durable protective immunity.

## Materials and methods

### Peptides

The preparation of B cell epitope from FMDV O/UK/11/2001, named “B” [VP1 protein (residues 140–158)], B cell epitope from FMDV C-S8c1, named “B-CS8” [VP1 protein (residues 136–154)], T-cell epitope 3A, named “T-3A” (3A protein, residues 21–35), B_2_ peptide (comprising 2 copies of “B”) and B_2_T dendrimer peptide (comprising 2 copies of “B” and one copy of “T-3A” linked via maleimide) has been previously described^34–36^ and is illustrated in Table 1.

### Virus

The serotype O FMDV stock O/UK/11/2001 (The Pirbright Institute, UK) was amplified and titrated in swine kidney cells (IBRS-2).

### Mice immunization

The experimental design and maintenance of the animals were in accordance with the current National and European regulations in animal welfare (EU Directive 86/609/EEC and Recommendation 2007/526/EC regarding the protection of animals used for experimental and other scientific purposes, enforced in Spanish law under Real Decreto 1201/2005). Accordingly, the experiments were conducted under the approval of CSIC Committee on Ethics of Animal Experiments and Biosafety, as well as of the National Committee on Ethics and Animal Welfare (PROEX 039.1/21). This study is reported in accordance with ARRIVE guidelines (https://arriveguidelines.org).

Groups of 5 to-6-week-old outbred female Swiss ICR1-CD1® mice (n = 5) were individually housed under standard conditions at the CBMSO animal facility. The mice, obtained from Envigo (Barcelona, Spain), were immunized subcutaneously, twice, on days 0 and 20 with a single 0.2 ml injection containing 100 µg of each peptide emulsified in Montanide ISA 50V2 (Seppic-France). Blood samples were collected by submandibular facial vein puncturing, to obtain serum for humoral response analyses, at 0, 20 and 32 dpi. At 32 dpi, mice were euthanized by initial asphyxiation in a CO_2_ chamber (fill rate of 40% displacement of the chamber volume per minute), followed by cervical dislocation of unconscious animals. The spleen, axillary draining lymph nodes and BM cells were harvested from euthanized mice for B and T-cell analyses. No anesthesia was administered to the animals prior to these procedures.

### Detection of anti-FMDV antibodies by ELISA

Specific antibodies were assayed by ELISA as described^34^ using plates coated with B peptide (1 µg) that were incubated with threefold dilutions of serum and detected using HRP-conjugated protein A. Plates were read at 450 nm and titers expressed as the reciprocal of the last serum dilution, given an absorbance range of 2 standard deviations above the background (serum on day 0) plus 2 SD.

### Virus neutralization test (VNT)

Neutralization assays were performed as previously described^34^. Briefly, serial twofold dilutions of each serum sample were incubated with 100 infection units—50% tissue culture infective doses (TCID_50_)—of FMDV O/UK/11/2001 for 1 h at 37 ºC. Next, a cell suspension of IBRS-2 cells (a swine kidney cell line obtained from Centro de Investigación en Sanidad Animal, CISA-INIA-CSIC, Spain) in DMEM was added and the plates were incubated for 48–72 h. End-point titers were calculated as the reciprocal of the final serum dilution that neutralized 100 TCID_50_ of homologous FMDV in 50% of the wells.

### Flow cytometry

For the analysis of lymphocyte populations, single cell suspensions were prepared by homogenized spleens and axillar lymph nodes using 70 µm nylon mesh cell strainer with plastic plunge in PBS 1X containing 2% FBS 2 mM EDTA. BM cells were extracted by centrifugation of punctured tibiae and femurs at up to 10.000 g for 10 s. Erythrocyte lysis was performed with ACK buffer (0.15 M NH_4_Cl, 10 mM KHCO_3_, 0.1 mM EDTA; pH = 7.2–7.4) by incubating spleen and BM 5 min at room temperature and washing with PBS 1X containing 2% FBS 2 mM EDTA. Single cell suspensions were incubated with FC block anti-CD16/32 (clone 93, Biolegend, San Diego, CA, USA) antibody to avoid non-specific surface antibody staining. Cells were incubated with the appropriate combination of the following antibodies: APC-Fire 810 B220 (clone RA3-6B2, BD Biosciences, Franklin Lakes, NJ, USA), PE CD138 (Clone 281-2, Biolegend), APC-Fire 750 IgD (clone 11-26c.2a, Biologend), AF488 GL7 (clone GL7, Biolegend,), PE-Cy7 CD95 (clone Jo2, BD Biosciences), BUV737 CD4 (clone RM4-4, BD Biosciences), v421 CD44 (clone IM7, Biolegend), AF488 CD62L (clone MEL-14, Biolegend), BV650 IgG1 (clone RMG1-1, Biolegend). Dead cells were excluded using fixable viability markers ghost-dye red 780 or ghost-dye violet 540 (TONBO Biosciences, San Diego, CA, USA). For intracellular staining of IgG, cells were fixed and permeabilized using BD Cytofix/Cytoperm™ Fixation/Permeabilization Kit (Cat No 554717) following the manufacture’s instructions and stained for 1 h with BV650 anti mouse-IgG1 at room temperature.

### IFN-γ ELISPOT

At 32 dpi, spleen cells were collected and analyzed for specific IFN-γ production by ELISPOT, as previously reported^36^. Briefly, 2.5 × 10^5^ splenocytes were shed in triplicate wells of Immobilon-P 96-well plates (Merck Millipore, Madrid, Spain) coated as reported with 5 µg/mL of mouse IFN-γ ELISPOT capture antibody (BD Biosciences). For the in vitro antigen recall, splenocytes from peptide-immunized mice were stimulated with 50 µg/mL of the peptide (B_2_T) used for mouse immunization or with T-3A peptide. As positive control, splenocytes were incubated with 10 µg/mL of phytohaemagglutinin-M (PHA-M, Sigma-Aldrich, Madrid, Spain) using cells incubated without antigen as negative control. After 48 h at 37 °C—5% CO_2_, plates were washed and incubated with 2 µg/mL of mouse IFN-γ ELISPOT detection antibody (BD Biosciences) followed by HRP-streptavidin (BD Biosciences). Antibody was visualized with 3-amino-9-ethyl carbazole (BD Biosciences). The frequency of peptide-specific T cells in the responding population was expressed as the mean number of spot-forming cells/10^6^ splenocytes, with background values (number of spots in negative control wells) subtracted from the respective counts of stimulated cells.

### B cell ELISPOT

At 32 dpi, BM cells were collected and analyzed for B cell ELISPOT to detect IgG1 ASCs. Briefly, Immobilon-P 96-well plates (Merck Millipore, Madrid, Spain) were coated, in triplicate wells, with different antigens (50 µg/ml): B_2_T, homologous B peptide and heterologous B peptide from FMDV C-S8c1 strain [VP1 (136–154)]^37^. Different amounts of BM cells (1.25 × 10^5^, 2.5 × 10^5^, 5 × 10^5^ and 1 × 10^6^ cells per well) were shed. After 18 h-incubation at 37 °C—5% CO_2_, plates were washed and incubated with 2 µg/mL of biotinylated rat anti-mouse IgG1 antibody (clone A85-1, BD Biosciences) followed by HRP-streptavidin (BD Biosciences). Antibody was visualized with 3-amino-9-ethyl carbazole (BD Biosciences). The frequency of peptide-specific B cells in the responding population was expressed as the mean number of spot-forming cells/10^6^ bone marrow cells, with background values (number of spots in negative control wells) subtracted from the respective counts of stimulated cells.

### Statistical analysis

Differences among peptide-immunized groups in FMDV-antibody titers and number of IFN-γ- or IgG1-producing cells were analyzed by unpaired two tail t-test, one-way ANOVA, followed by Tukey’s post-hoc comparisons tests, or two-way ANOVA, followed by Uncorrected Fisher’s LSD post-hoc comparison tests. Each analysis was properly indicated in the figure legend. All *p* values are two sided, and *p* values < 0.05 were considered significant. Statistical analyses were conducted using GraphPad Prism Software 10.0 (San Diego, CA, USA).

## Data Availability

All data generated or analyzed during this study are included in this published article.

## Declarations

### Biosafety and biosecurity statement

All work involving live FMDV was carried out at the Centro de Investigación en Sanidad Animal (CISA; INIA-CSIC), a facility authorised under Spanish and European legislation (EU Regulations 2016/429 and 2017/625) for in vivo and in vitro handling of FMDV. CISA operates a dedicated BSL3 laboratory for FMDV research that complies with EU biosecurity standards.
